# Global Burden of Small Vessel Disease–Related Brain Changes on MRI Predicts Cognitive and Functional Decline

**DOI:** 10.1161/STROKEAHA.119.026170

**Published:** 2019-11-08

**Authors:** Hanna Jokinen, Juha Koikkalainen, Hanna M. Laakso, Susanna Melkas, Tuomas Nieminen, Antti Brander, Antti Korvenoja, Daniel Rueckert, Frederik Barkhof, Philip Scheltens, Reinhold Schmidt, Franz Fazekas, Sofia Madureira, Ana Verdelho, Anders Wallin, Lars-Olof Wahlund, Gunhild Waldemar, Hugues Chabriat, Michael Hennerici, John O’Brien, Domenico Inzitari, Jyrki Lötjönen, Leonardo Pantoni, Timo Erkinjuntti

**Affiliations:** 1 From the Clinical Neurosciences, Neurology, University of Helsinki and Helsinki University Hospital (H.J., H.M.L., S. Melkas, T.E.), Finland; 2 Department of Psychology and Logopedics, Faculty of Medicine (H.J., H.M.L.), Finland; 3 Medical Imaging Center, Radiology, University of Helsinki and Helsinki University Hospital (A.K.), Finland; 4 Combinostics, Ltd, Finland (J.K., T.N., J.L.); 5 VTT Technical Research Centre of Finland (J.K., J.L.); 6 Faculty of Health Sciences, University of Eastern Finland (J.K.); 7 Department of Radiology, Medical Imaging Center, Tampere University Hospital, Finland (A.B.); 8 Biomedical Image Analysis Group, Department of Computing, Imperial College London, United Kingdom (D.R.); 9 Department of Radiology and Nuclear Medicine (F.B.), Neuroscience Campus Amsterdam, VU University Medical Center, the Netherlands; 10 Alzheimer Center and Department of Neurology (P.S.), Neuroscience Campus Amsterdam, VU University Medical Center, the Netherlands; 11 Institutes of Neurology and Healthcare Engineering, University College London, United Kingdom (F.B.); 12 NIHR Biomedical Research Centre at University College London Hospitals NHS Foundation Trust, University College London, United Kingdom (F.B.); 13 Department of Neurology, Medical University of Graz, Austria (R.S., F.F.); 14 Department of Neurosciences, Santa Maria Hospital, University of Lisbon, Portugal (S. Madureira, A.V.); 15 Sahlgrenska Academy, Institute of Neuroscience and Physiology, Section for Psychiatry and Neurochemistry, University of Gothenburg, Sweden (A.W.); 16 Department of Neurobiology, Care Sciences and Society, Division of Clinical Geriatrics, Karolinska Institutet, Sweden (L.-O.W.); 17 Department of Neurology, Danish Dementia Research Centre, Rigshospitalet, University of Copenhagen, Denmark (G.W.); 18 Department of Neurology, Hopital Lariboisiere, APHP and INSERM U1161–University Denis Diderot (DHU NeuroVasc), France (H.C.); 19 Medical Faculty Mannheim, University of Heidelberg, Germany (M.H.); 20 Department of Psychiatry, University of Cambridge, United Kingdom (J.O.); 21 Institute of Neuroscience, Italian National Research Council (D.I.); 22 Department NEUROFARBA, University of Florence, Italy (D.I.); 23 Department of Neuroscience and Biomedical Engineering, School of Science, Aalto University, Finland (J.L.); 24 L. Sacco Department of Biomedical and Clinical Sciences, University of Milan, Italy (L.P.).

**Keywords:** brain, cerebral small vessel diseases, humans, image processing, computer assisted, neuropsychology

## Abstract

Cerebral small vessel disease is characterized by a wide range of focal and global brain changes. We used a magnetic resonance imaging segmentation tool to quantify multiple types of small vessel disease–related brain changes and examined their individual and combined predictive value on cognitive and functional abilities.

**Methods—:**

Magnetic resonance imaging scans of 560 older individuals from LADIS (Leukoaraiosis and Disability Study) were analyzed using automated atlas- and convolutional neural network–based segmentation methods yielding volumetric measures of white matter hyperintensities, lacunes, enlarged perivascular spaces, chronic cortical infarcts, and global and regional brain atrophy. The subjects were followed up with annual neuropsychological examinations for 3 years and evaluation of instrumental activities of daily living for 7 years.

**Results—:**

The strongest predictors of cognitive performance and functional outcome over time were the total volumes of white matter hyperintensities, gray matter, and hippocampi (*P*<0.001 for global cognitive function, processing speed, executive functions, and memory and *P*<0.001 for poor functional outcome). Volumes of lacunes, enlarged perivascular spaces, and cortical infarcts were significantly associated with part of the outcome measures, but their contribution was weaker. In a multivariable linear mixed model, volumes of white matter hyperintensities, lacunes, gray matter, and hippocampi remained as independent predictors of cognitive impairment. A combined measure of these markers based on *Z* scores strongly predicted cognitive and functional outcomes (*P*<0.001) even above the contribution of the individual brain changes.

**Conclusions—:**

Global burden of small vessel disease–related brain changes as quantified by an image segmentation tool is a powerful predictor of long-term cognitive decline and functional disability. A combined measure of white matter hyperintensities, lacunar, gray matter, and hippocampal volumes could be used as an imaging marker associated with vascular cognitive impairment.

## Abstract 2

Supplemental Digital Content is available in the text.

Cerebral small vessel disease (SVD) is a frequent cause of stroke and the primary subtype of vascular cognitive impairment. The neuroimaging features of SVD include small subcortical infarcts, lacunes, white matter hyperintensities (WMH), enlarged perivascular spaces (EPVS), microbleeds, and brain atrophy,^1^ which have been variably associated with cognitive performance.^2–7^

Recently, SVD has been considered as a dynamic whole brain disease because of the diffuse nature, common microvascular pathologies, and varying progression of its lesion types.^8^ A multifactorial approach, taking comprehensive imaging data into consideration with clinical follow-up, may be optimal in characterizing the course of SVD. Visual scoring systems for combinations of magnetic resonance imaging (MRI) findings have been introduced to represent the total load of SVD^9^ and cerebrovascular disease.^10^ These scales are pragmatic but limited in sensitivity. Since the accumulating brain changes in SVD are on a continuum, it is worthwhile to investigate global lesion load with continuous measures rather than ordinal scales.

Computer-generated MRI segmentation has become available for different types of SVD changes. Brain volume, WMH, and infarcts have received the most attention, while fewer methods have been presented to quantify lacunes and EPVS. This study aimed to find an optimal combination of the major SVD-related brain changes by using MRI quantification tools. We examined volumetric data of WMH, lacunes, EPVS, chronic cortical infarcts, and global and regional brain volume to identify the measures with the strongest associations with cognitive decline. A combined continuous measure was constructed to represent the global burden of brain changes, and its predictive value was validated against long-term cognitive and functional outcome.

## Methods

The data that support the findings of this study are available from the corresponding author on reasonable request.

### Participants and Design

The data were drawn from LADIS (Leukoaraiosis and Disability Study)—a longitudinal multicenter collaboration investigating the role of age-related WMH in functional disability.^11^ In total, 639 subjects, aged 65 to 84 years, were enrolled at 11 European centers. The reasons for referral and the inclusion-exclusion criteria are detailed in the online-only Data Supplement. At baseline, the subjects were classified to have mild-to-severe WMH on MRI according to the modified Fazekas scale.^11^ All subjects were independent or had no more than minimal impairment in instrumental activities of daily living (IADL) as evaluated with the Lawton IADL scale.^12^

At baseline (2001–2003), the subjects underwent brain MRI and clinical assessments including a standard neurological examination, functional status evaluation, and a neuropsychological examination. In follow-up, the clinical and neuropsychological assessments were repeated annually over 3 years. The number of subjects with clinical evaluation was 639 at baseline, 582 at the first, 554 at the second, and 523 at the third follow-up visit. The number of subjects with neuropsychological assessment was 638, 569, 496, and 468, respectively.

A prolonged follow-up ≤7 years (2008–2009) was carried out by telephone interviews collecting data from the proxy/informant with the IADL scale focusing on activities in the past 3 months. Conversion from functional independence into disability was defined as an increase of IADL score from 0 or 1 to ≥2. Of the initial 639 subjects, 94 subjects had died during follow-up. In total, data of outcome in terms of functional disability or death were available for 633 (99%) subjects.

Ethical approval was given by the local ethics committees of each center. Written informed consent was received from all participants.

### MRI Acquisition

Brain MRI was administered to the subjects according to the same protocol at each center. The sequences included T1-weighted 3-dimensional magnetization-prepared rapid acquisition gradient-echo, T2-weighted fast-spin echo, and fluid-attenuated inversion recovery images as described in the online-only Data Supplement.^13–15^

### Image Analysis

**Figure 1.**
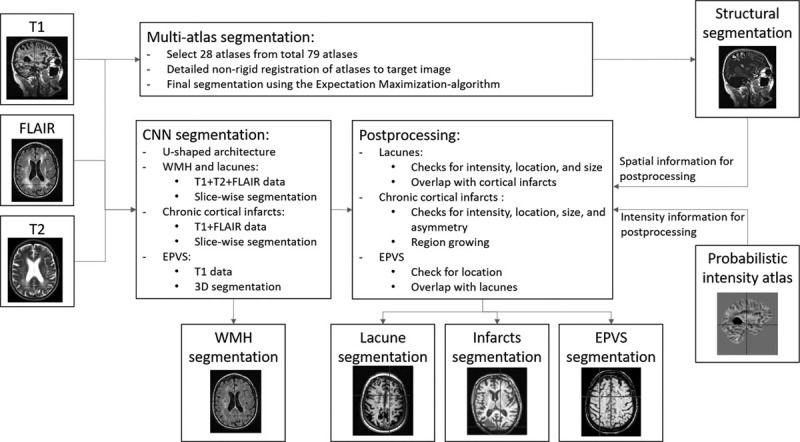
Flowchart of the image analysis methods (detailed description given in the online-only Data Supplement). 3D indicates 3 dimensional; EPVS, enlarged perivascular space; FLAIR, fluid-attenuated inversion recovery; and WMH, white matter hyperintensities.

**Figure 2.**
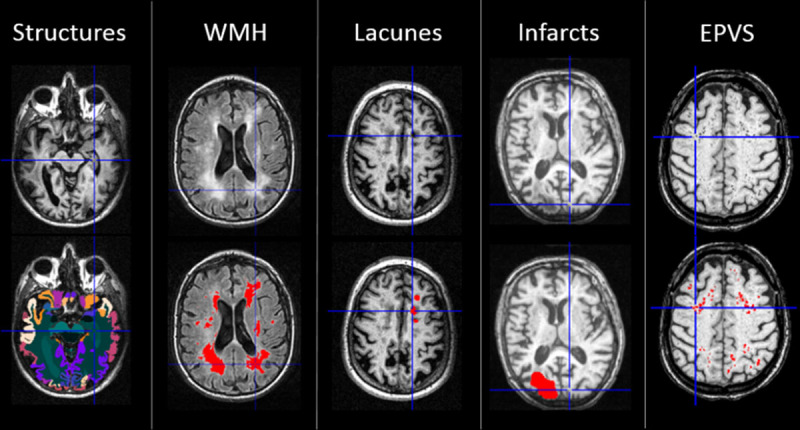
Examples of the segmentation results for brain structures, white matter hyperintensities (WMH), lacunes, cortical infarcts, and enlarged perivascular spaces (EPVS). **Top**, Original T1/fluid-attenuated inversion recovery images. **Bottom**, Segmentation results overlaid with color on the original images.

Of the original 639 subjects, 78 cases were excluded from this study because of incomplete set of MRI sequences and 1 case because of failed multimodal registration in preprocessing. The image analysis methods and their validation results are presented in detail in the online-only Data Supplement. An overview of the image analysis strategy is given in Figure 1 and examples of the segmentation results in Figure 2. The evaluation included WMH, lacunes, EPVS, and brain atrophy based on the STRIVE (Standards for Reporting Vascular Changes on Neuroimaging) neuroimaging guidelines.^1^ Chronic cortical infarcts were also taken into consideration as occasional concomitant findings in SVD and potential contributors to clinical outcome. However, microbleeds and recent subcortical infarcts were not evaluated because of unavailable susceptibility and diffusion-weighted imaging data.

WMH, lacunes, infarcts, and EPVS were segmented using U-shaped convolutional neural networks on fluid-attenuated inversion recovery and T2 and T1 sequences as outlined in Figure 1. The segmentation was performed using 10-fold cross-validation. The ground truth segmentations required for training were generated using manual and semiautomatic methods. An automated postprocessing of the convolutional neural networks results was performed to remove possible erroneous segmentations utilizing spatial information from the multi-atlas segmentation and intensity information from a probabilistic intensity atlas. In addition, the overlap of the segmentations was checked. Volumes of the brain structures were measured from T1 images using an automated image quantification tool (Combinostics, Ltd, Finland; www.cneuro.com/cmri/), by which the brain is segmented into 133 regions with a multi-atlas method. First, 28 best-matching atlases were selected from the original 79 manually segmented atlases (http://www.neuromorphometrics.com/), and the selected atlases were nonrigidly registered with the T1 image. The brain segmentation was generated from the 28 atlas segmentations using the expectation-maximization algorithm. The present analysis comprised volumes of total brain tissue, cerebral gray matter (GM), white matter, lobar regions, and hippocampi. All volumes were normalized for intracranial volume using the method described by Buckner et al.^16^

### Neuropsychological Assessment

The LADIS cognitive test battery included the Mini-Mental State Examination, the Vascular Dementia Assessment Scale–Cognitive Subscale (VADAS), the Stroop Test, and the Trail Making Test.^17^ Mini-Mental State Examination and VADAS total scores were used as measures of global cognitive function. Cognitive subdomains of processing speed, executive functions, and memory were evaluated with psychometrically robust compound scores detailed in the online-only Data Supplement.

### Statistical Analyses

The predictive value of each MRI measure on the 4 cognitive scores in 3-year follow-up was investigated individually with linear mixed modeling (restricted maximum likelihood estimation, unstructured covariance structure) to allow for incomplete data in follow-up. After ruling out multicollinearity, the measures with the strongest associations with cognitive performance in terms of main effects and MRI×time interactions (indicating a change over time) and surviving familywise Bonferroni correction for multiple comparisons were entered simultaneously in a multivariable linear mixed model. The measures with significant independent contributions to cognition were then combined as equal components into a single score of global SVD-related brain changes by averaging the standardized *Z* scores of each volumetric measure. Finally, the significance of the individual and combined MRI measures in predicting poor functional outcome was analyzed with Cox proportional hazards models.

All analyses were adjusted for age, sex, years of education, and study center. The models with multiple MRI predictors were rerun by additionally controlling for hypertension and diabetes mellitus, and for incident stroke during follow-up. Since all results remained unchanged, these analyses were not reported. A log transformation was applied for WMH, lacunes, cortical infarcts, and EPVS volumes to account for nonnormality of the distributions.

## Results

### Sample Characteristics

The subjects with complete MRI segmentation data (n=560) did not differ from the excluded subjects in age, sex, years of education, WMH Fazekas score, or baseline Mini-Mental State Examination score (*P*>0.05). The characteristics of the sample are shown in Table 1.

**Table 1.**
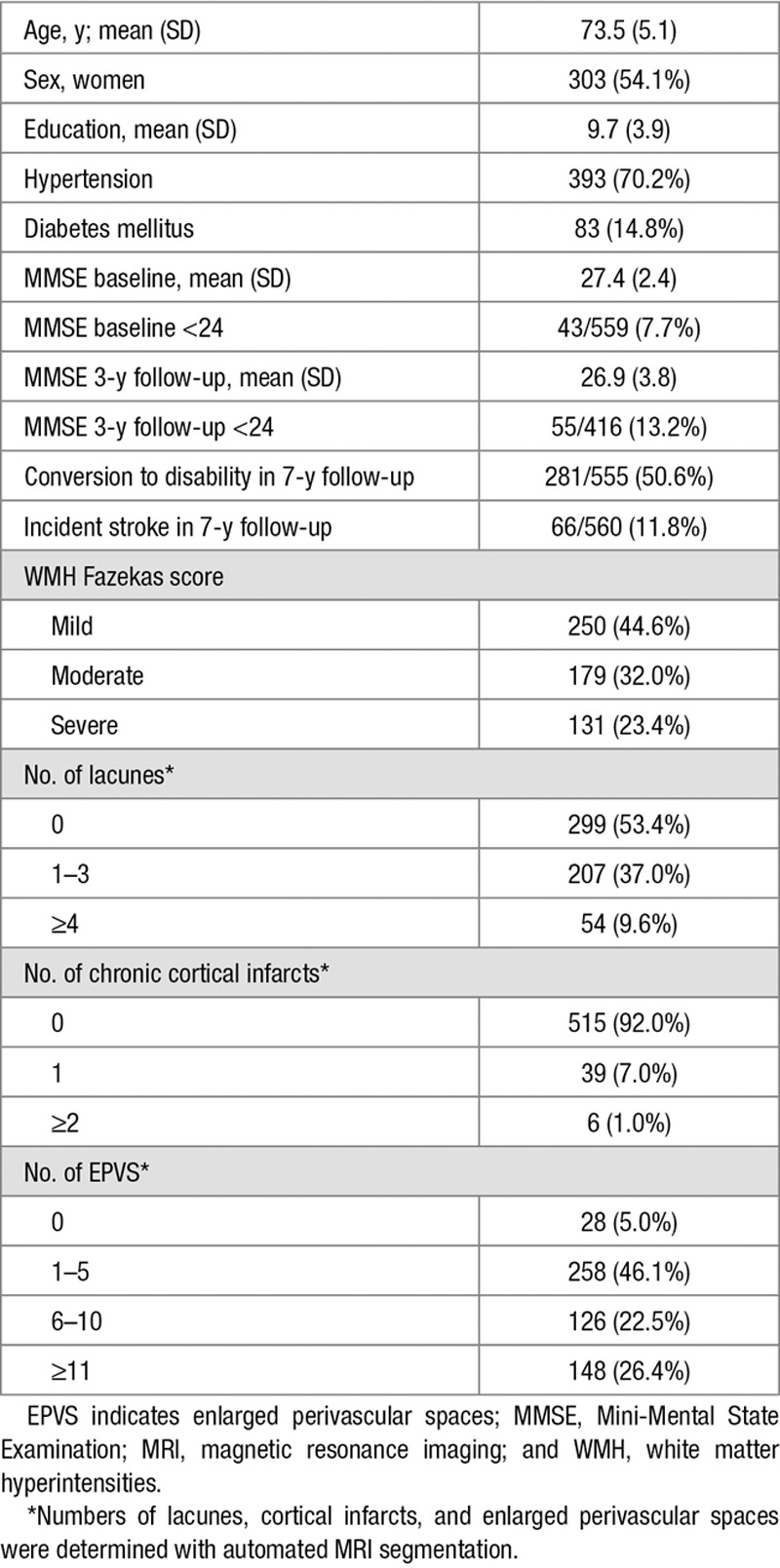
Characteristics of the Study Sample (n=560)

### MRI Segmentation Results

The MRI segmentation results of total and regional lesion volumes and brain structures are shown in Table I in the online-only Data Supplement and correlations between the main MRI predictors in Table II in the online-only Data Supplement.

### Individual Associations Between the MRI Measures and Cognition

The associations between individual MRI volumes and cognitive scores in 3-year follow-up as investigated with linear mixed models are presented in Table 2. After adjustments for age, sex, education, and center, all WMH volumes were significantly associated with worse overall performance in all cognitive scores (main effect) and with steeper rate of decline (interaction with time) in processing speed, executive functions, and VADAS total score. Total, periventricular, and anterior WMH tended to have slightly stronger relationships with cognitive scores compared with the other white matter regions, although the differences between regions were modest.

**Table 2.**
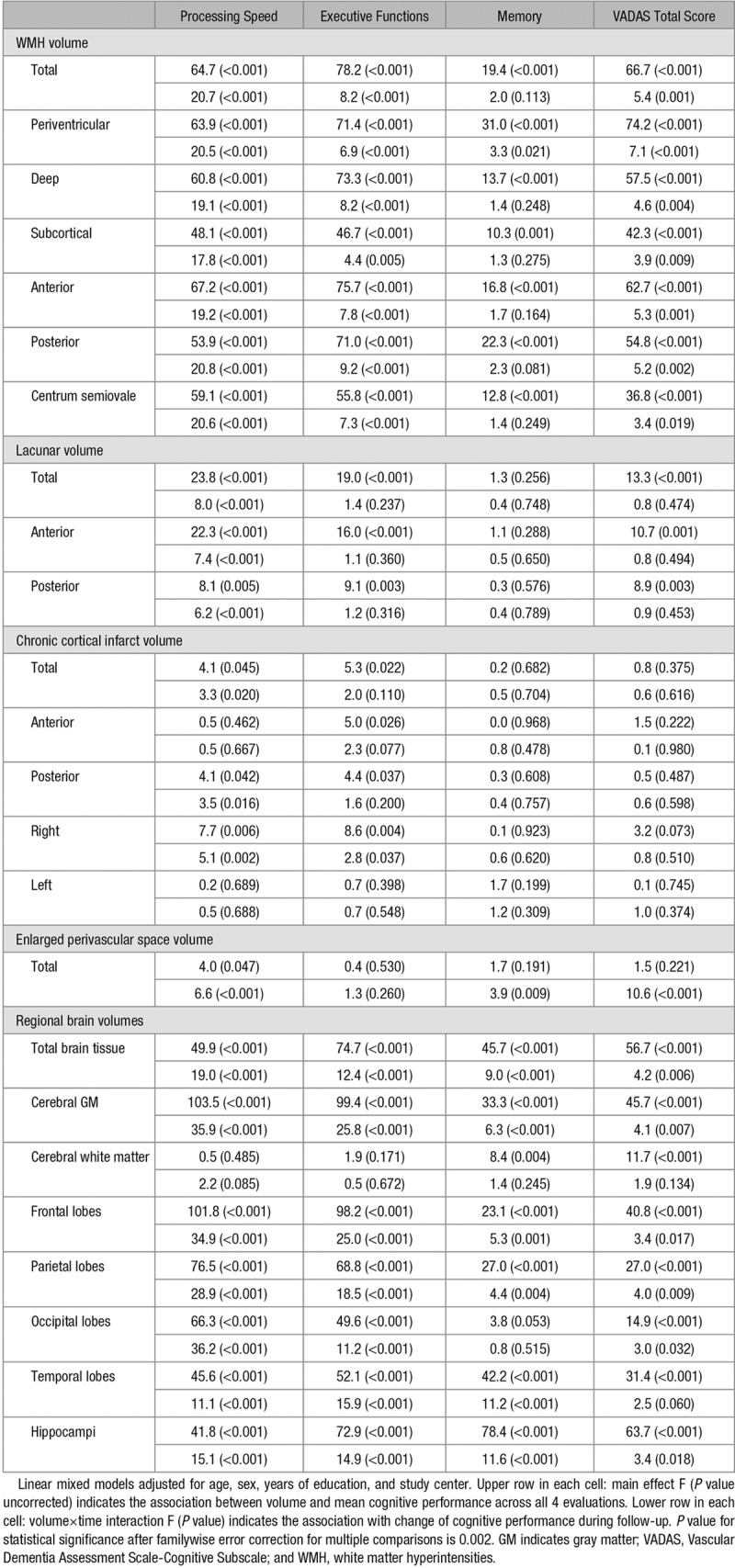
Individual Predictive Values of Magnetic Resonance Imaging Measures on Cognitive Functions in 3-Year Follow-Up

Total and regional lacunar volumes predicted poorer overall performance in processing speed, executive functions and VADAS, and steeper decline in processing speed, independently of confounders. However, none of the lacunar volumes significantly predicted the memory domain score. Anterior volumes had slightly more prominent effects on cognition than those of the posterior regions.

Chronic cortical infarcts had relatively low predictive value on cognitive performance. Total and regional infarct volumes predicted weakly the overall level and change in processing speed and executive functions. Higher infarct volume was associated with negative main effect on cognitive performance, but there was variability in the estimates of change per follow-up year (data not shown), and the relationships did not survive correction for multiple comparisons.

Total EPVS volume was significantly associated with overall performance and decline in processing speed, as well as decline in memory and VADAS total score.

Total and regional brain volumes showed strong associations with decline in all cognitive scores. Total cerebral GM and frontal lobe volumes were the strongest predictors of speed and executive functions, whereas hippocampal volume most prominently predicted memory and VADAS total score.

### Independent Contributions of the MRI Measures on Cognition

Based on the above-described relationships with cognitive functions (Table 2), total volumes of WMH, lacunes, EPVS, cerebral GM, and hippocampi were selected and entered together in a linear mixed model adjusted for confounders. Cortical infarcts were left out at this stage because of their weak associations with cognitive decline. As shown in Table 3, total WMH volume significantly predicted all 4 cognitive scores independently of the other lesion types. Lacunar volume independently predicted executive functions. Cerebral GM volume was associated with processing speed, whereas hippocampal volume with VADAS total score, executive functions, and memory (Table 3). EPVS had no independent contribution to any of the cognitive measures.

**Table 3.**
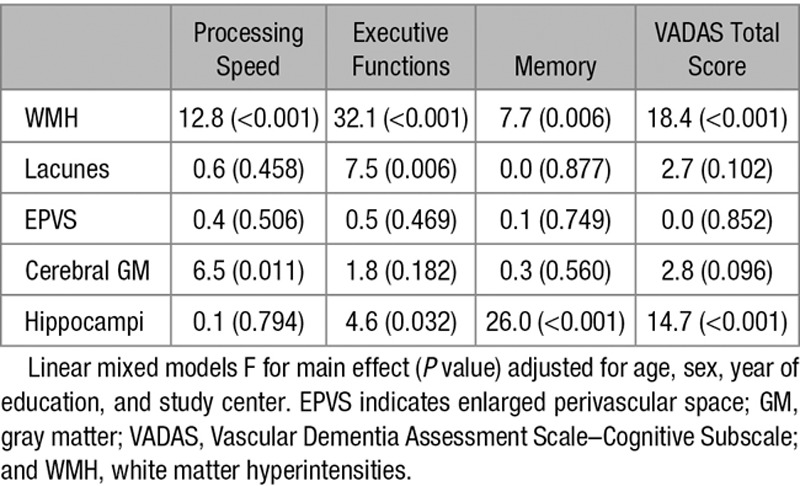
Combined Models for Global Small Vessel Disease Burden: Independent Significance of the Magnetic Resonance Imaging Predictors on Overall Cognitive Performance Over 3-Year Follow-Up

### Global Score of SVD-Related Brain Changes as a Predictor of Cognitive Functions

A quantitative global measure of SVD-related brain changes was constructed by taking the mean of the standardized MRI measures (*Z* scores) significant in the multivariable models above, that is, total volumes of WMH, lacunes, cerebral GM, and hippocampi. The scales of GM and hippocampal volumes were first inversed so that higher scores reflected higher degree of brain changes for all variables. This global score proved to have strong and consistent associations with poorer overall level of performance and steeper rate of decline across all cognitive scores, surpassing the effects of the individual volumes. In linear mixed models identical to individual analyses, the global score significantly predicted processing speed (main effect F 120.6, *P*<0.001; interaction with time F 37.3, *P*<0.001), executive functions (F 148.3, *P*<0.001; F 21.5, *P*<0.001), memory (F 54.4, *P*<0.001; F 7.4, *P*<0.001), and the VADAS total score (F 101.2, *P*<0.001; F 5.1, *P*=0.002, respectively).

### Associations Between MRI Measures and Functional Outcome

Poor functional outcome was defined as subject’s transition to disability or death within the prolonged 7-year follow-up period. As investigated with individual Cox regression analyses controlling for the confounders, poor outcome was significantly predicted by total WMH (HR, 4.2 [95% CI, 2.9–5.9]; *P*<0.001), lacunar (HR, 8.1 [95% CI, 3.0–21.7]; *P*<0.001), cerebral GM (HR, 0.990 [95% CI, 0.986–0.993]; *P*<0.001), and hippocampal volumes (HR, 0.57 [95% CI, 0.49–0.67]; *P*<0.001) but not by EPVS or cortical infarct volumes (*P*>0.05). In a multivariable model of the 4 significant MRI measures, WMH (HR, 2.6 [95% CI, 1.7–3.8]; *P*<0.001), lacunar (HR, 3.2 [95% CI, 1.1–9.4]; *P*=0.034), and hippocampal volumes (HR, 0.67 [95% CI, 0.55–0.81]; *P*<0.001) remained as independent predictors. Finally, the combined score of SVD-related brain changes was significantly related to functional outcome independently of the confounders (HR, 2.7 [95% CI, 2.2–3.3]; *P*<0.001).

## Discussion

Thus far, there has been no agreed procedure for how to assess the different types of brain changes related to cerebral SVD. In the current study, we leveraged the wealth of information provided by convolutional neural networks- and atlas-based brain MRI segmentation methods to characterize the diverse pathologies of SVD in older individuals. The volumes with the highest associations with long-term cognitive and functional outcome were identified, and the core features were combined to represent the global burden of SVD-related brain changes. The results showed that the combined measure was a more powerful predictor of cognitive decline than the individual measures alone.

When considered in isolation, total WMH and GM volumes had the strongest relationships with both global and domain-specific cognitive performance over a 3-year follow-up. Lacunar volumes were significantly related to global cognitive function, processing speed, and executive functions but not with memory. Anterior regions had slight predominance over posterior regions in predicting cognitive functions possibly reflecting a dysfunction of frontal-subcortical networks. Of the regional brain volumes, hippocampal volume also proved to have strong associations with cognitive scores, most prominently with global cognitive function and memory domain. However, EPVS and chronic cortical infarct volumes had weaker associations with cognitive decline. In the multivariable models of multiple MRI measures, total WMH, lacunar, GM, and hippocampal volumes remained as independent predictors of cognitive functions over a 3-year follow-up.

The individual contributions of the MRI segmentation volumes are in line with earlier studies using either visual scales or volumetric measures. Within the LADIS study, cognitive impairment has been related to WMH Fazekas score^14^ and semiautomatic volumetry,^4,5^ number and location of lacunes,^4,15^ and visual ratings of global and medial temporal lobe atrophy.^5^ The decisive role of WMH, lacunes, and brain atrophy in cognitive decline of SVD has been confirmed by several other studies.^3,6,18^ However, despite being a frequent imaging finding in SVD, EPVS have not invariably predicted cognitive decline.^2,3^

The selection of MRI features for the global measure of brain changes was based on the significance of associations between volumes and multiple longitudinal cognitive and functional outcomes. As the relationship of individual volumes varied between different outcomes, the selected components were given equal weight in the combined score. Importantly, brain atrophy as reflected by reduced global and regional GM volumes was considered together with vascular lesions. Our earlier study has revealed synergistic interactions between atrophy and vascular changes on cognitive decline.^5^ In SVD, WMH and lacunes are closely related to GM atrophy and whole brain volume.^19,20^ In particular, hippocampal and medial temporal lobe atrophy are associated with SVD and contribute to cognitive impairment also in the absence of Alzheimer pathology.^21,22^ Brain atrophy is certainly not related to pure SVD only, as concomitant neurodegenerative processes are promoted by SVD. In our study, the mechanisms behind hippocampal atrophy remain unclear because specific biomarkers of concomitant early Alzheimer disease were unavailable. More important than to disentangle these processes etiologically, however, is their obvious contribution to predicting cognitive decline and disability.

The current study is one of the first to analyze the clinical significance of automated MRI segmentation features of multiple types of SVD-related brain changes simultaneously. The visual composite score suggested by Staals et al^9^ has been found to correlate with general cognitive ability^23^ and decline in executive functions,^24^ but the results have not been consistent.^25^ For the visual SVD score, the different lesion types are evaluated using dichotomous ratings. Continuous volumetric measures are, therefore, expectedly more sensitive in detecting global brain changes. Recently, a computer-generated global measure of whole brain atrophy and vascular disease has been proposed and shown to have a stronger relationship with cognition as compared with WMH volume alone and visual total SVD score.^26^ Another study has also reported that a combination of SVD features contributed more to cognitive performance after stroke than the individual measures.^27^ These studies have been limited by relatively small sample sizes and brief cognitive measures. Hippocampal volume has not been included in the measures of global SVD burden before.

Among the strengths of our study are the large and well-characterized sample of subjects, a novel image analysis approach achieving high accuracy in lesion quantification, and the longitudinal design. We found a close correspondence between the estimates of brain changes detected with the present segmentation method and those obtained with manual delineations. Specifically, WMH volume was highly equivalent to the semiautomatic WMH analysis taken as the ground truth.^13^ Lacunes, cortical infarcts, and EPVS also reached good accuracy as compared with expert manual delineations (online-only Data Supplement). We applied detailed neuropsychological evaluations to yield psychometrically sound compound indices for both global cognitive function and specific domains. The assessment was repeated annually over 3 years, while the evaluation of functional outcome in terms of IADL was extended ≤7 years. The associations were independent of age, sex, education, study center, and main vascular risk factors.

As the subjects were recruited in clinical settings based on WMH found on brain imaging, the sample represents a mixed population of cases with different degrees of SVD, but the results cannot be directly generalized to other elderly populations. Because of unavailable susceptibility-weighted MRI sequences, we could not include cerebral microbleeds in our analyses, although they are regarded as part of the typical SVD neuroimaging features^1^ and have been identified as risk factors for cognitive dysfunction.^7^ Nor were we able to differentiate recent subcortical infarcts or cerebral microinfarcts. Although the present study provides thus far one of the most extensive quantifications of multiple types of imaging features, the method is still not all-inclusive and thus calls for further research.

The imaging data used in this study were relatively heterogeneous multicenter data representing the normal clinical variability in image quality. The contrast of some images was fairly low, and the slice thickness of the T2 and fluid-attenuated inversion recovery sequences was 5 mm (T1, 1 mm). Consequently, the smallest EPVS and lacunes were not clearly visible. Both lacunes and EPVS often have similar appearance in T1 images, which may cause mix-up of the segmentations. As the LADIS sample was recruited based on age-related WMH, the number of cases with cortical infarcts was relatively small (in total 73 infarcts), and the training set for the convolutional neural networks remained restricted. Utilization of high-resolution and high-quality imaging data and larger training set would improve segmentation accuracy. Further studies with advanced imaging technologies are required to confirm the predictive value of these lesion types on cognitive decline.

Various automated image quantification tools are becoming part of clinical practice. Although there is a great promise of making image interpretation more objective and consistent, users should also understand limitations of such tools. Visual inspection is necessary to verify the results so that possible errors can be taken into account in interpretation. In the present study, normalization for intracranial volume was done indirectly based on registrations to a reference template, which may introduce bias as compared with normalization with actual intracranial volume.

Another limitation of our study is a possible attrition bias in follow-up neuropsychological data, because subjects with more severe decline are more likely to drop out. Linear mixed models were used as the statistical approach to allow for incomplete observations and utilize all available data in the longitudinal setting.

## Conclusions

By a robust MRI segmentation method capable of identifying multiple types of imaging features of cerebral SVD and brain atrophy, we showed that WMH, lacunar, cerebral GM, and hippocampal volumes had the greatest independent predictive value for both cognitive and functional outcome in older individuals with ≤7-year follow-up. A combined continuous measure of these 4 imaging findings was highly predictive of cognitive decline, surpassing the contributions of the individual cerebrovascular disease biomarkers. Global quantification of SVD-related brain changes provides a comprehensive neuroimaging metric associated with vascular cognitive impairment and may be desirable for intervention studies as a more valid surrogate than single MRI findings.

## Sources of Funding

LADIS (Leukoaraiosis and Disability Study) was supported by the European Union (grant QLRT-2000-00446). This work has received funding from the European Union Seventh Framework Programme for research, technological development, and demonstration under grant agreement No. 611005 (PredictND) and 601055 (VPH-DARE@IT), and Tekes–the Finnish Funding Agency for Technology and Innovation (4171/31/2017 DeepBrain project).

## Disclosures

Dr Jokinen reports grants from Helsinki University Hospital governmental funding for clinical research during the conduct of the study. Dr Koikkalainen reports grants from European Commission and Tekes–Finnish Funding Agency for Technology and is a shareholder at Combinostics, Ltd. Dr Rueckert reports grants from European Commission during the conduct of the study and personal fees from Heartflow and CIRSE Cardiovascular outside the submitted work. Dr Barkhof reports a grant from Research Councils UK (RCUK), personal fees from Biogen, Roche, Lundbeck, and IXICO and grants from Novartis outside the submitted work. Dr Chabriat reports personal fees from Hovid Company and Servier outside the submitted work. J. O’Brien reports personal fees from TauRx, Eisai, GE Healthcare, and Axon; grants and personal fees from Avid/Lilly; and grants from Alliance Medical outside the submitted work. Dr Lötjönen reports grants from European Commission and Tekes–Finnish Funding Agency for Technology, lecture fees from Merck and Sanofi, and is a shareholder at Combinostics, Ltd. The other authors report no conflicts.

## Supplementary Material
