# Supplementary material for: Global Burden of Small Vessel Disease–Related Brain Changes on MRI Predicts Cognitive and Functional Decline
Source: Stroke. 2019 Nov 8;51(1):170–8. doi: 10.1161/STROKEAHA.119.026170 (PMC6924941; doi:10.1161/STROKEAHA.119.026170)
Supplement: Supplementary file 1 [file str-51-170-s001.pdf]

SUPPLEMENTAL MATERIAL

Global burden of small vessel disease-related brain changes on MRI predicts cognitive and functional decline

Hanna Jokinen, PhD; Juha Koikkalainen, PhD; Hanna M. Laakso, MA; Susanna Melkas, MD, PhD; Tuomas Nieminen, MSc; Antti Brander MD, PhD; Antti Korvenoja, MD, PhD; Daniel Rueckert, PhD; Frederik Barkhof, MD, PhD; Philip Scheltens, MD, PhD; Reinhold Schmidt, MD; Franz Fazekas, MD; Sofia Madureira, PsyD; Ana Verdelho, MD, PhD; Anders Wallin, MD, PhD; Lars-Olof Wahlund, MD, PhD; Gunhild Waldemar, MD, PhD; Hugues Chabriat, MD, PhD; Michael Hennerici, MD; John O'Brien, DM; Domenico Inzitari, MD; Jyrki Lötjönen, PhD; Leonardo Pantoni, MD, PhD; Timo Erkinjuntti, MD, PhD

Table of contents

Page

Supplemental methods

Referral reasons and inclusion-exclusion criteria 2

MRI protocol 2

MRI segmentation method 2

Ground truth segmentations 2

Image analysis 3

White matter hyperintensities, lacunes, infarcts and perivascular spaces 3

Volumetry of anatomical regions 4

Brain-size normalization 4

Regions of Interest 4

Validation of image analysis 5

Neuropsychological evaluation 5

References 5

Supplemental tables

Table I. MRI segmentation volumes 7

Table II. Correlations among the normalized lesion volumes and structural volumes 8

Supplemental Appendix

Leukoaraiosis and Disability Study: List of participating centers and initial personnel 9

SUPPLEMENTAL METHODS

Referral reasons and inclusion-exclusion criteria

The Leukoaraiosis and Disability (LADIS) study is a multinational collection and follow-up of initially nondisabled elderly subjects with age-related white matter hyperintensities (WMH).[1] The subjects were referred to the study based on complaints including mild cognitive (26%) or motor disturbances (4%), minor cerebrovascular events (19%), mood alterations (2%) and other neurological problems (20%). Subjects in whom WMH were incidentally found on brain imaging (17%) as well as volunteers participating as controls in other studies (11%) were also included.

The inclusion criteria were: a) age 65-84 years, b) WMH on MRI of any degree according to the a revised version of the Fazekas scale,[1] c) no or mild disability as evaluated with the Instrumental Activities of Daily Living scale[2] (no impairment at all or only 1 item compromised), d) presence of a regularly contactable informant, e) agreement to sign an informed consent. The exclusion criteria were: a) subject likely to drop out because of the presence of severe illnesses (cardiac, hepatic or renal failure, cancer or other relevant systemic diseases), b) severe unrelated neurological diseases, c) leukoencephalopathy of nonvascular origin (immunological demyelinating, metabolic, toxic, infectious, other), e) severe psychiatric disorders, f) inability to give an informed consent, g) inability or refusal to undergo cerebral MRI.

MRI protocol

All subjects were studied by MRI following a standard protocol. The scans were collected centrally at the Image Analysis Centre of the VU University Medical Center, Amsterdam, the Netherlands. The protocol included the following sequences: T1-weighted 3D MPRAGE (magnetization prepared rapid-acquisition gradient-echo, scan parameters: coronal or sagittal plane, field of view [FOV] 250 mm, matrix 256×256 or 512×512, slice thickness: 1 mm [isotropic voxels], TE: 2 to 7 ms, TR: 9 to 26 ms, FA 10% to 30%), T2-weighted FSE (fast spin echo, scan parameters: axial plane, FOV 250 mm, matrix 256×256 or 512×512, slice thickness: 5 mm, interslice gap 0.5 mm, TE: 100 to 130 ms, TR: 4000 to 8000 ms), and FLAIR (fluid-attenuated inversion recovery, scan parameters: axial plane, FOV 250 mm, matrix 256×256 or 512×512, slice thickness: 5 mm, interslice gap 0.5 mm, TE: 100 to 160 ms, TR: 6000 to 10000 ms, TI: 2000 to 2400).[3-5]

MRI segmentation method

Ground truth segmentations

The automated image analysis methods used in this study required ground truth segmentations for training. These ground truth segmentations were generated using manual and semi-automatic methods described below.

WMH, lacunes and EPVS were determined on the basis of the neuroimaging guidelines of the STandards for ReportIng Vascular changes on nEuroimaging (STRIVE).[6] Hyperintense areas in the white matter on FLAIR sequences without cavitation were regarded as WMH of presumed vascular origin. Lacunes were defined as round or ovoid subcortical fluid-filled cavities (signal similar to CSF) of 3-15 mm in diameter usually having a hyperintense rim in FLAIR sequences. Lacunes were distinguished from EPVS by the difference in size (EPVS generally less than 3 mm in diameter) and shape (EPVS often linear without hyperintense rim). Chronic cortical infarcts typically related to large-vessel disease were also included in the analyses as occasional concomitant findings in SVD and defined as the necrotic tissue located on the cortex (hypointense in T1 and FLAIR sequences). Due to unavailable susceptibility and diffusion weighted imaging data, microbleeds and recent subcortical infarcts were not evaluated.

The ground truth segmentation for WMH was obtained as a part of the initial analysis of the LADIS study, where WMH was semi-automatically defined on the axial FLAIR images in periventricular,

subcortical and infratentorial regions.[3] The segmentation was based on a seed-growing technique with local thresholding, where the lesions were marked and borders were set on each slice. Areas of hyperintensity around infarctions and lacunes were omitted.

The ground truth segmentation for lacunes and chronic cortical infarcts were drawn manually from co-registered T1, T2, and FLAIR images using an in-house software tool developed for the manual segmentation of images. All the segmentations were confirmed by an experienced neuroradiologist. In total, there were lacunes in 220 scans (655 lacunes) and cortical infarcts in 55 scans (73 infarcts). The ground truth segmentations for EPVS (53 scans, 2759 EPVS) were drawn manually from T1 images using an in-house software tool.

Image analysis

White matter hyperintensities, lacunes, infarcts and perivascular spaces

WMH, lacunes, EVPS and chronic cortical infarcts were segmented using U-shaped convolutional neural networks (CNN).[7,8] CNN are machine learning models that take large number of training samples as an input and build a model that will predict the output based on the training samples. In this study, the input data was the different MRI sequences (T1/T2/FLAIR) and the output data was the ground truth segmentations.

The CNN architecture used in this work was the U-shaped residual network presented by Guerrero et al.[7] In short, the network consists of 12 layers with about 1M parameters. There are 8 residual elements, 3 deconvolutional layers, and final convolutional layer that gives the class probabilities for each voxel as an output.

Three CNN were trained:

1. CNN for the simultaneous segmentation of WMH and lacunes utilizing T1, T2, and FLAIR images as input data. Because of the large slice thickness (5 mm) of the T2 and FLAIR images the segmentation was performed using two-dimensional data for each slice separately. The network was trained using image patches (64x64 voxels).
2. CNN for the slice-wise segmentation of cortical infarcts using T1 and FLAIR images as input data.
3. CNN for the segmentation of EPVS using only T1 image as input data. The segmentation was performed using full three-dimensional data, where the network was trained using 64x64x32 voxel patches.

The training of the CNN models was performed using 10-fold cross-validation, i.e., 90% of the dataset was used in training and the rest 10% in testing. This was repeated 10 times so that each image was once used in test set. Because only a small portion of the images included lacunes or infarcts, the training of the CNN was performed by emphasizing the training samples with these lesion types. The CNN for EPVS segmentation was trained using the subset with ground truth segmentations.

Due to the relatively small training set and large slice thickness, it was noticed that false positive segmentations of lacunes and cortical infarcts occurred. For example, parts of sulci or ventricles could be miss-classified as lacunes in a single 2D slice. Therefore, an automated post-processing step was developed to adjust the segmentations to accurately correspond with the STRIVE guidelines.[6] In order to remove segmentations from erroneous regions, spatial information on the location of the segmentations was obtained from the multi-atlas segmentation results.[9] On the other hand, a probabilistic atlas of grey-scale values (generated from 534 subjects without major vascular pathologies) was used to provide comparison data for the intensities.

Following postprocessing steps were performed:

1. Discard lacunes if:
    a. diameter is smaller than 3 mm
    b. is located inside ventricles or sulci
    c. no clear cavity on T1 sequence
    d. coincides with a cortical infarct segmentation
2. Discard cortical infarct if:
    a. volume is too small
    b. is not located on cortex
    c. is not atypically dark in T1
    d. not enough asymmetry in T1 intensities between hemispheres
    e. does not have hyperintensity surrounding in FLAIR
3. Perform region growing for cortical infarcts by applying dilation five times and adding voxels that have atypically low intensity in T1
4. Discard EPVS if:
    a. is located inside sulci or ventricles
    b. coincides with a lacune segmentation.

Volumetry of anatomical regions

Volumes of the brain structures were measured from T1 images using an automated image quantification tool (Combinostics Ltd., Tampere, Finland, www.cneuro.com/cmri/).[10] This tool segments the brain into 133 regions (102 cortical parcellations and 31 sub-cortical regions) using a multi-atlas segmentation method based on 79 manually segmented atlases (http://www.neuromorphometrics.com/).[9] In short, the T1 image of a subject and the atlases are registered using coarse non-rigid deformation. Then, an atlas selection is used to select the 28 best-matching atlases out of the 79 atlases for more detailed non-rigid registration. A probabilistic atlas, generated from these atlas segmentations, is used as a prior in the intensity-based classification using the Expectation-Maximization algorithm producing the final brain segmentation.

Brain-size normalization

All volumes were normalized for intracranial volume using the method where a loose-fitting brain mask of the subject is registered to a corresponding template image and a scaling factor is defined.[11]

Regions of Interest

In addition to the total WMH/lacune/cortical infarct volumes, following regional volumes were computed: periventricular, deep white matter, subcortical, anterior and posterior, left and right hemisphere, and centrum semiovale. The left-right division and the deep white matter, subcortical, and periventricular regions were defined in the subject space based on the multi-atlas segmentation results. The anterior-posterior division and the centrum semiovale were first defined in the Montreal Neurological Institute space (MNI 152-template). The anterior-posterior separation was done using the slice y=110. The centrum semiovale was defined by first extracting the white matter superior to the lateral ventricles (z>32), and then the sulcal white matter regions were removed using a set of morphological operations. The regions of interest were then propagated to the subject images based on a sequence of registrations (MNI – reference template – subject image).

The structural volumetry measures evaluated in this study were the volumes of total brain tissue, cerebral grey matter (GM), cerebral white matter, hippocampi as well as frontal, parietal, occipital and temporal lobes. These volumes were obtained as combinations of the original 133 brain regions of the multi-atlas segmentation.

Validation of image analysis

Correlation between the WMH volumes of ground truth and automated segmentations was 0.98, and the mean Dice similarity coefficient was 0.77. The corresponding correlation for the volume of lacunes was 0.79, for the volume of chronic cortical infarcts 0.83 and for the volume of EPVS 0.91. The method for the segmentation of structural volumes has been validated in Lötjönen et al.[9] where, for example, the correlation for the volume of hippocampus was 0.94.

Neuropsychological evaluation

The cognitive test battery of the LADIS study comprised the Mini-Mental State Examination (MMSE),[12] the Vascular Dementia Assessment Scale–Cognitive Subscale (VADAS),[13] the Stroop test and the Trail making test.[14] Global cognitive function was evaluated with the total scores of MMSE and VADAS.

For the evaluation of cognitive subdomains, three compound measures were constructed by averaging the *z* scores of individual tests within each domain.[15] The scales were first inverted, where necessary, so that higher scores indicated better performance in all variables. Specifically, processing speed was evaluated with the Trail making part A, VADAS Maze task and Digit cancellation. Executive functions were assessed with the Stroop III-II time difference score, Trail making B-A time difference score, VADAS Symbol digit modalities test and Verbal fluency. Memory was evaluated with the VADAS Immediate word recall, Delayed recall, Word recognition and Digit span subtests. The 3-factor model has been supported by confirmatory factor analysis study suggesting that the domains are valid latent variables of cognitive performance and relatively consistent over time.[16]

Within the present sample (subjects with complete MRI data, n=560), VADAS total score was available for 532 of 559 subjects (95%) participating the baseline neuropsychological assessment and 379 of 416 subjects (91%) participating in the 3rd-year follow-up assessment. The respective numbers were 546 (98%) and 394 (95%) for processing speed, 514 (92%) and 364 (88%) for executive functions, and 554 (99%) and 396 (95%) for memory.

References

(1) Pantoni L, Basile AM, Pracucci G, Asplund K, Bogousslavsky J, Chabriat H, et al. Impact of age-related cerebral white matter changes on the transition to disability -- the LADIS study: rationale, design and methodology. *Neuroepidemiology*. 2005;24:51-62.

(2) Lawton MP, Brody EM. Assessment of older people: self-maintaining and instrumental activities of daily living. *Gerontologist*. 1969;9:179-186.

(3) van Straaten EC, Fazekas F, Rostrup E, Scheltens P, Schmidt R, Pantoni L, et al. Impact of white matter hyperintensities scoring method on correlations with clinical data: the LADIS study. *Stroke*. 2006;37:836-840.

(4) van der Flier, W M, van Straaten EC, Barkhof F, Verdelho A, Madureira S, Pantoni L, et al. Small vessel disease and general cognitive function in nondisabled elderly: the LADIS study. *Stroke*. 2005;36:2116-2120.

(5) Benisty S, Gouw AA, Porcher R, Madureira S, Hernandez K, Poggesi A, et al. Location of lacunar infarcts correlates with cognition in a sample of non-disabled subjects with age-related white-matter changes: the LADIS study. *J Neurol Neurosurg Psychiatry*. 2009;80:478-483.

(6) Wardlaw JM, Smith EE, Biessels GJ, Cordonnier C, Fazekas F, Frayne R, et al. Neuroimaging standards for research into small vessel disease and its contribution to ageing and neurodegeneration. *Lancet Neurol*. 2013;12:822-838.

(7) Guerrero R, Qin C, Oktay O, Bowles C, Chen L, Joules R, et al. White matter hyperintensity and stroke lesion segmentation and differentiation using convolutional neural networks. *Neuroimage Clin*. 2017;17:918-934.

(8) Goodfellow I, Bengio Y, Courville A. Deep learning. Cambridge, MA: MIT Press; 2016.

(9) Lötjönen JM, Wolz R, Koikkalainen JR, Thurfjell L, Waldemar G, Soininen H, et al. Fast and robust multi-atlas segmentation of brain magnetic resonance images. *Neuroimage*. 2010;49:2352-2365.

(10) Koikkalainen J, Rhodius-Meester H, Tolonen A, Barkhof F, Tijms B, Lemstra AW, et al. Differential diagnosis of neurodegenerative diseases using structural MRI data. *Neuroimage Clin*. 2016;11:435-449.

(11) Buckner RL, Head D, Parker J, Fotenos AF, Marcus D, Morris JC, et al. A unified approach for morphometric and functional data analysis in young, old, and demented adults using automated atlas-based head size normalization: reliability and validation against manual measurement of total intracranial volume. *Neuroimage*. 2004;23:724-738.

(12) Folstein MF, Folstein SE, McHugh PR. "Mini-mental state". A practical method for grading the cognitive state of patients for the clinician. *J Psychiatr Res*. 1975;12:189-198.

(13) Ferris SH. General measures of cognition. *Int Psychogeriatr*. 2003;15 Suppl 1:215-217.

(14) Lezak MD, Howieson DB, Bigler ED, Tranel, D. Neuropsychological assessment. 5th ed. New York: Oxford University Press; 2012.

(15) Madureira S, Verdelho A, Ferro J, Basile AM, Chabriat H, Erkinjuntti T, et al. Development of a neuropsychological battery for the Leukoaraiosis and Disability in the Elderly Study (LADIS): experience and baseline data. *Neuroepidemiology*. 2006;27:101-116.

(16) Moleiro C, Madureira S, Verdelho A, Ferro JM, Poggesi A, Chabriat H, et al. Confirmatory factor analysis of the Neuropsychological Assessment Battery of the LADIS study: a longitudinal analysis. *J Clin Exp Neuropsychol*. 2013;35:269-278.

SUPPLEMENTAL TABLES

Supplemental table I. Automated MRI segmentation volumes normalized for intracranial volume (ml)

	Mean (SD)
White matter hyperintensities	
Total	19.3 (19.4)
Periventricular	4.9 (3.2)
Deep	12.2 (14.8)
Subcortical	2.0 (2.4)
Anterior	12.9 (13.6)
Posterior	6.4 (7.1)
Centrum semiovale	2.8 (4.2)
Lacunes	
Total	0.22 (0.45)
Anterior	0.19 (0.41)
Posterior	0.03 (0.09)
Chronic cortical infarcts	
Total	0.34 (1.79)
Anterior	0.05 (0.62)
Posterior	0.29 (1.56)
Right	0.13 (0.94)
Left	0.05 (0.63)
Enlarged perivascular spaces	
Total	0.28 (0.65)
Regional brain volumes	
Brain tissue, total	1099.8 (50.0)
Cerebral grey matter, total	483.3 (53.5)
Cerebral white matter, total	415.9 (64.3)
Frontal lobe, right	89.8 (11.2)
Frontal lobe, left	90.4 (11.0)
Temporal lobe, right	53.9 (7.4)
Temporal lobe, left	55.2 (6.7)
Parietal lobe, right	49.5 (6.4)
Parietal lobe, left	49.1 (6.3)
Occipital lobe, right	33.3 (5.5)
Occipital lobe, left	31.5 (5.9)
Hippocampus, right	3.1 (0.5)
Hippocampus, left	3.0 (0.5)

Supplemental table II. Correlations among the normalized lesion volumes and structural volumes

	Lacunes	Cortical infarcts	EPVS	Cerebral grey matter	Hippocampus
WMH	0.20 (<0.001)	0.15 (<0.001)	0.01 (0.798)	-0.41 (<0.001)	-0.42 (<0.001)
Lacunes		0.08 (0.058)	0.10 (0.024)	-0.11 (0.012)	-0.03 (0.461)
Cortical infarcts			-0.1 (0.789)	-0.09 (0.045)	-0.06 (0.161)
EPVS				-0.03 (0.449)	0.00 (0.965)
Cerebral grey matter					0.67 (<0.001)

Pearson correlation coefficient (p value)

EPVS indicates enlarged perivascular spaces; WMH, white matter hyperintensities

SUPPLEMENTAL APPENDIX

Leukoaraiosis and Disability Study: List of participating centers and initial personnel

Helsinki, Finland (Neurology, Helsinki University Hospital and University of Helsinki, Finland): Timo Erkinjuntti, MD, PhD, Tarja Pohjasvaara, MD, PhD, Pia Pihanen, MD, Raija Ylikoski, PhD, Hanna Jokinen, PhD, Meija-Marjut Somerkoski, MPsych, Riitta Mäntylä, MD, PhD, Oili Salonen, MD, PhD; Graz, Austria (Department of Neurology and Department of Radiology, Division of Neuroradiology, Medical University Graz): Franz Fazekas, MD, Reinhold Schmidt, MD, Stefan Ropele, PhD, Brigitte Rous, MD, Katja Petrovic, MagPsychol, Ulrike Garmehi, Alexandra Seewann, MD; Lisboa, Portugal (Serviço de Neurologia, Centro de Estudos Egas Moniz, Hospital de Santa Maria): José M. Ferro, MD, PhD, Ana Verdelho, MD, Sofia Madureira, PsyD, Carla Moleiro, PhD; Amsterdam, The Netherlands (Department of Radiology and Neurology, VU Medical Center): Philip Scheltens, MD, PhD, Ilse van Straaten, MD, Frederik Barkhof, MD, PhD, Alida Gouw, MD, Wiesje van der Flier, PhD; Goteborg, Sweden (Institute of Clinical Neuroscience, Goteborg University): Anders Wallin, MD, PhD, Michael Jonsson, MD, Karin Lind, MD, Arto Nordlund, PsyD, Sindre Rolstad, PsyD, Ingela Isblad, RN; Huddinge, Sweden (Karolinska Institutet, Department of Neurobiology, Care Sciences and Society; Karolinska University Hospital Huddinge): Lars-Olof Wahlund, MD, PhD, Milita Crisby, MD, PhD, Anna Pettersson, RPT, PhD, Kaarina Amberla, PsyD; Paris, France (Department of Neurology, Hopital Lariboisiere): Hugues Chabriat, MD, PhD, Karen Hernandez, psychologist, Annie Kurtz, psychologist, Dominique Hervé, MD, Sarah Benisty, MD, Jean Pierre Guichard, MD; Mannheim, Germany (Department of Neurology, University of Heidelberg, Klinikum Mannheim): Michael Hennerici, MD, Christian Blahak, MD, Hansjorg Baezner, MD, Martin Wiarda, PsyD, Susanne Seip, RN; Copenhagen, Denmark (Memory Disorders Research Group, Department of Neurology, Rigshospitalet, and the Danish Research Center for Magnetic Resonance, Hvidovre Hospital, Copenhagen University Hospitals): Gunhild Waldemar, MD, DMSc, Egill Rostrup, MD, MSc; Charlotte Ryberg, MSc, Tim Dyrby MSc, Olaf B. Paulson, MD, DMSc; Ellen Garde, MD, PhD; Kristian Steen Frederiksen, MD; Newcastle-upon-Tyne, UK (Institute for Ageing and Health, Newcastle University): John O'Brien, DM, Sanjeet Pakrasi, MRCPsych, Mani Krishnan MRCPsych, Andrew Teodorczuk, MRCPsych, Michael Firbank, PhD, Philip English, DCR, Thais Minett, MD, PhD.

The Coordinating center is in Florence, Italy (Department of Neurological and Psychiatric Sciences, University of Florence): Leonardo Pantoni, MD, PhD, Domenico Inzitari, MD (Study Coordinators); Luciano Bartolini, PhD, Anna Maria Basile, MD, PhD, Eliana Magnani, MD, Monica Martini, MD, Mario Mascalchi, MD, PhD, Marco Moretti, MD, Anna Poggesi, MD, PhD, Giovanni Pracucci, MD, Emilia Salvadori, PhD, Michela Simoni, MD.

The LADIS Steering Committee is formed by Domenico Inzitari, MD, Timo Erkinjuntti, MD, PhD, Philip Scheltens, MD, PhD, Marieke Visser, MD, PhD, and Peter Langhorne, MD, BSC, PhD, FRCP who replaced in this role Kjell Asplund, MD, PhD beginning in 2005.
